# The Global Infectious Diseases Epidemic Information Monitoring System: Development and Usability Study of an Effective Tool for Travel Health Management in China

**DOI:** 10.2196/24204

**Published:** 2021-02-16

**Authors:** Dayong Gu, Jianan He, Jie Sun, Xin Shi, Ying Ye, Zishuai Zhang, Xiangjun Wang, Qun Su, Wenjin Yu, Xiaopeng Yuan, Ruiling Dong

**Affiliations:** 1 Department of Laboratory Medicine, Shenzhen Second People's Hospital The First Affiliated Hospital of Shenzhen University Health Science Center Shenzhen China; 2 Shenzhen International Travel Health Care Center (Shenzhen Customs District Port Outpatient Clinics) Shenzhen Customs District Shenzhen China; 3 Shenzhen Academy of Inspection and Quarantine Shenzhen Customs District Shenzhen China; 4 Business School, All Saints Campus Manchester Metropolitan University Manchester United Kingdom; 5 Department of Statistics & Applied Probability National University of Singapore Singapore Singapore; 6 Harbin Institute of Technology, Shenzhen Shenzhen China; 7 Shenzhen Datathinking Corporation Shenzhen China; 8 Department of Laboratory Medicine ZhuJiang Hospital Southern Medical University Guangzhou China

**Keywords:** infectious disease, epidemic information, travel health, global, surveillance

## Abstract

**Background:**

Obtaining comprehensive epidemic information for specific global infectious diseases is crucial to travel health. However, different infectious disease information websites may have different purposes, which may lead to misunderstanding by travelers and travel health staff when making accurate epidemic control and management decisions.

**Objective:**

The objective of this study was to develop a Global Infectious Diseases Epidemic Information Monitoring System (GIDEIMS) in order to provide comprehensive and timely global epidemic information.

**Methods:**

Distributed web crawler and cloud agent acceleration technologies were used to automatically collect epidemic information about more than 200 infectious diseases from 26 established epidemic websites and Baidu News. Natural language processing and in-depth learning technologies have been utilized to intelligently process epidemic information collected in 28 languages. Currently, the GIDEIMS presents world epidemic information using a geographical map, including date, disease name, reported cases in different countries, and the epidemic situation in China. In order to make a practical assessment of the GIDEIMS, we compared infectious disease data collected from the GIDEIMS and other websites on July 16, 2019.

**Results:**

Compared with the Global Incident Map and Outbreak News Today, the GIDEIMS provided more comprehensive information on human infectious diseases. The GIDEIMS is currently used in the Health Quarantine Department of Shenzhen Customs District (Shenzhen, China) and was recommended to the Health Quarantine Administrative Department of the General Administration of Customs (China) and travel health–related departments.

**Conclusions:**

The GIDEIMS is one of the most intelligent tools that contributes to safeguarding the health of travelers, controlling infectious disease epidemics, and effectively managing public health in China.

## Introduction

In the past, when infectious disease outbreaks have occurred in certain countries, such as the Middle East Respiratory Syndrome (MERS) outbreak in South Korea in 2015, specific health examinations had to be performed on travelers from those countries upon entering China [1]. This kind of health management work at Chinese ports of entry are undertaken by travel health officers (referred to as health quarantine officers in China) from China Customs. This is one of the key approaches to preventing and controlling the transmission of infectious diseases [2].

Along with the development of global economic integration, the number of international travelers to China is gradually increasing. For instance, Shenzhen is an international metropolis in China, with an urban population of more than 10 million [3]. In 2019, approximately 242 million passengers passed through the Port of Shenzhen [4]. Travel health officers at China Customs use a temperature monitoring system and epidemiological investigation as the main detecting tools to identify infected travelers [5]. Many travelers may not be aware of their infection status while traveling [6]. Therefore, travel health officers must pay attention to the latest epidemic information regarding certain infectious diseases to determine whether these travelers may be infected and prepare the related public health materials. In general, information concerning global infectious disease outbreaks is manually collected, which is a time-consuming and error-prone process. Travel health officers require an infectious disease information collection system that can automatically collect epidemic information from a large number of websites, extract key information, and translate it into the native language. For these reasons, the Harbin Institute of Technology (Shenzhen, China) and Datathinking Corporation (Shenzhen, China) developed the Global Infectious Diseases Epidemic Information Monitoring System (GIDEIMS) in coordination with the Central Laboratory of Health Quarantine of the Shenzhen International Travel Health Care Center (Shenzhen Customs District Port Outpatient Clinics). The GIDEIMS is currently used by the Health Quarantine Department at the Port of Shenzhen Customs, and the system was recommended to the Health Quarantine Administrative Department of the General Administration of Customs (China) and travel health–related departments. An Epidemic Information Team has been established by the General Administration of Customs (China) aimed at supporting the travel health officers and related departments in public health management. With the application of the GIDEIMS, updated and useful global infectious disease information is sent to the concerned parties daily, as well as useful information for infectious disease prevention and control, such as instructions on how to prepare public health materials, including masks and test kits. The aim of the GIDEIMS is to provide travelers and travel health staff with a helpful tool for public health management.

## Methods

### Selection of Websites

Web queries on infectious diseases could be one of the most accurate, cost-effective, and labour-extensive sources of syndromic surveillance [7]. Some established and/or official websites are frequently used by related staff and citizens to gain human infectious disease epidemic information. The websites used in the GIDEIMS are listed in Table 1; most infectious disease epidemic information, including information about COVID-19, can be found on these websites. Although all of these websites show excellent performance in the search and distribution of epidemic information, they are characterized by specific limitations. For instance, ProMED [8] reports on human diseases, as well as plant and animal diseases, while the Global Incident Map reports on fewer than 40 types of human infectious diseases, excluding chicken pox. Moreover, the epidemic information provided by the World Health Organization is reported by the member of states, and its distribution may be delayed. Data from different resources must be generated to obtain comprehensive information; the combined expertise of the different systems enhances performance for the early detection of infectious disease outbreaks [9]. Considering that the traditional manual search approach is time-consuming and may be inaccurate, as well as the fact that most of the existing websites are only offered in non–Chinese languages, Chinese travel health officers require a user-friendly infectious disease system that can effectively translate diverse information from multiple sources. For these purposes, we developed the GIDEIMS to provide a simple, effective, and sustainable tool for obtaining information on global human infectious disease epidemics.

**Table 1:** Websites used in the Global Infectious Diseases Epidemic Information Monitoring System.

Website number	Name and responsible department	Important column
1^a^	WHO^b^ [10]	“Disease Outbreak News”
2^a^	WHO Western Pacific Region [11]	“Outbreaks and emergencies” in “Emergencies”
3^a^	WHO Regional Office for the Eastern Mediterranean [12]	
4^a^	WHO Regional Office for Europe [13]	“Emergencies” from “Health topics”
5^a^	WHO Regional Office for the Americas [14]	Epidemiological Alerts and Updates
6^a^	WHO Regional Office for Africa [15]	Outbreaks and other emergencies
7^a^	ECDC^c^ [16]	News & events
8^a,d^	MOH^e^ (Kingdom of Saudi Arabia) [17]	Command and control center
9^a,d^	Travel Health Service, Department of Health, Hong Kong, China [18]	“Travel Health News”
10^a^	CDC^f^ (US) [19]	“Outbreaks”
11^a,d^	MOH (Singapore) [20]	“Weekly Infectious Diseases Bulletin” in “disease update”
12^a^	Global Polio Education Initiative [21]	“This week” in “Polio Today”
13^g^	Program for Monitoring Emerging Diseases, International Society for Infectious Diseases [22]	Need to distinguish human or animal diseases
14^a,d^	Centre for Health Protection, Department of Health (Hong Kong, China) [23]	“Outbreaks”
15^a,d^	CDC (South Korea) [24]	“Domestic Infectious Disease Occurrence” from “Archives”
16^a,d^	Department of Disease Control (Thailand) [25]	Weekly Disease Forecast
17^g^	Outbreak News Today, satellite of The Global Dispatch Inc [26]	Recent posts
18^g^	Global Incident Map [27]	50 newest events
19^a^	Pan American Health Organization [28]	“Epidemiological Alerts and Updates”
20^a^	Travel health notices, Government of Canada [29]	“Travel health notices”
21^g^	Center for Infectious Disease Research and Policy, Academic Health Center, University of Minnesota (MN, US) [30]	“News and Perspective”
22^a,d^	CDC, MOH (Taiwan) [31]	Professional and public version
23^a,d^	Unit for Communicable Disease Prevention and Diseases Surveillance, CDC (Macau, China) [32]	“latest news”
24^a,d^	Chinese National Influenza Center [33]	“Weekly Report”
25^g^	HealthMap, Harvard University (MA, US) [34]	“Outbreaks Near Me”
26^a,d^	Nigeria Center for Disease Control and Prevention [35]	“Weekly Epidemiological Report” from “Publications”

^a^Official public websites.

^b^WHO: World Health Organization.

^c^ECDC: European Centre for Disease Prevention and Control.

^d^Users pay close attention to epidemic information from this area.

^e^MOH: Ministry of Health.

^f^CDC: Centers for Disease Control and Prevention.

^g^This website gives comprehensive and timely epidemic information.

Meanwhile, several research organizations and government departments have their own information systems (eg, the Global Public Health Intelligence Network from the Public Health Agency of Canada). These websites were not used in the GIDEIMS due to their limitations or unavailability in China [36-40] (Table 2). At present, the GIDEIMS automatically collects information concerning more than 200 infectious diseases from 26 established official epidemic websites and Baidu news [41]. Web crawlers, which are used to retrieve information from websites and can be modified according to the requirements of the user, provide an advanced technique for information searching [42].

**Table 2:** Established and/or official public websites not used in the Global Infectious Diseases Epidemic Information Monitoring System.

Website number	Name and responsible department	Limitations
1	Medisys, The European Union [43]	Some information on global infectious diseases is delayed (eg, Ebola from Democratic Republic of the Congo).
2	Biocaster, National Institute of Informatics (Japan) [7,8]	This project ran from 2012 to 2016; currently unavailable.
3	Epispider, CDC^a^ (US) [7,9]	Currently unavailable from the mentioned website address and CDCa (US) websites.
4	Project Argus, Georgetown University (Washington, DC, US) and MITRE Corporation (VA, US), sponsored by the Government of the US [7,36]	Currently unavailable from the mentioned website address.
5	The RODS^b^ Open Source Project, RODS Library, University of Pittsburgh (PA, US) [9]	The software requires purchasing a license, and the website has not been updated since 2009.
6	GPHIN^c^, Public Health Agency of Canada [36,37]	Currently unavailable in the websites of the Public Health Agency of Canada. The WHO^d^ website contains the main information collected by the GPHIN [37].
7	Google Inc [44]	The same visitor is not allowed to visit the site frequently, Chinese government also has restrictions on access to Google.

^a^CDC: Centers for Disease Control and Prevention.

^b^RODS: Real-time Outbreak and Disease Surveillance.

^c^GPHIN: Global Public Health Intelligence Network.

^d^WHO: World Health Organization.

### Structure

The GIDEIMS uses distributed web crawler [45,46] and cloud acceleration technologies to automatically collect epidemic information. Natural language processing and in-depth learning technologies are used to intelligently process epidemic information collected in 28 languages. The language translation service is provided by Baidu Translate, which is recognized as one of most reliable online translation services in China [47]. Cloud acceleration technology is used to improve work performance of the system.

**Figure 1:**
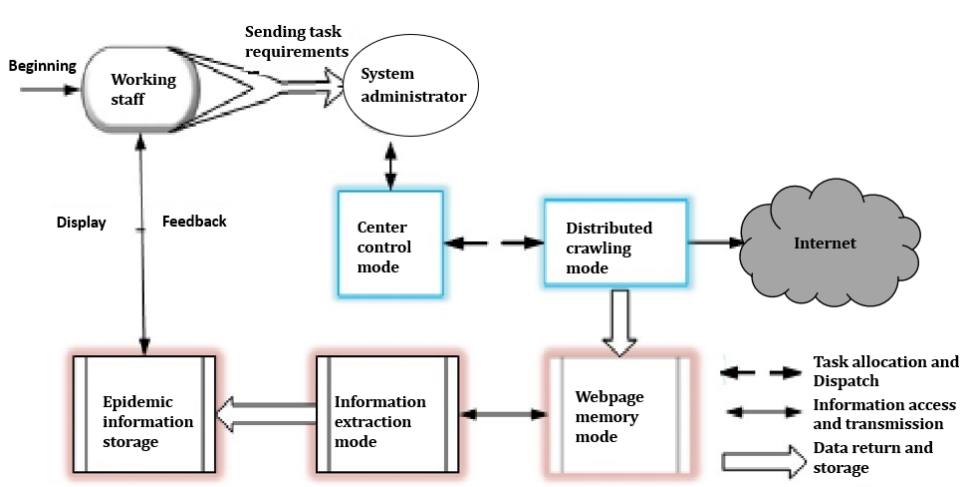
Principle of the Global Infectious Diseases Epidemic Information Monitoring System design.

Figure 1 shows the principle of the design of the GIDEIMS. In the figure, working staff refers to travel health officers; they provided the addresses/names of the epidemic information websites and requirements to the GIDEIMS administrator. The GIDEIMS administrator operated the center control mode according to the requirements set by the working staff. Subsequently, a series of webpage crawling, information extraction, and processing operations were performed.

The center control mode is the core mode of the entire system. It includes the setup of the crawling strategy, assignment of tasks, and management, testing, and debugging of the system by the administrator of the GIDEIMS.

The distributed crawling mode is the execution mode of the system and the key to determining the efficiency of the entire system. In this mode, multiple crawling machines cooperate to find webpages on the internet and jointly complete the crawling task.

The main task of the webpage memory mode is to find webpages on the internet using the crawler mode and store them in a particular structure. The main task of the information extraction mode is to define extraction rules according to acquisition tasks set by the user and webpage characteristics. Moreover, this mode extracts the information available on the webpage identified by the crawler mode according to the rules and transmits the extracted results to the storage mode.

The purpose of the epidemic information storage mode is to develop a data table related to the extraction rules defined by the information extraction mode.

## Results

### Main Functions of the GIDEIMS

The GIDEIMS includes 7 functions: (1) GIDEIMS map, (2) latest outbreaks, (3) epidemic inquiry, (4) epidemic information entry, (5) general analysis, (6) basic setup, and (7) further functions.

#### GIDEIMS Map

The GIDEMS map shows the latest available information worldwide and in China. Information is acquired and shown automatically by the virtual private network.

**Figure 2:**
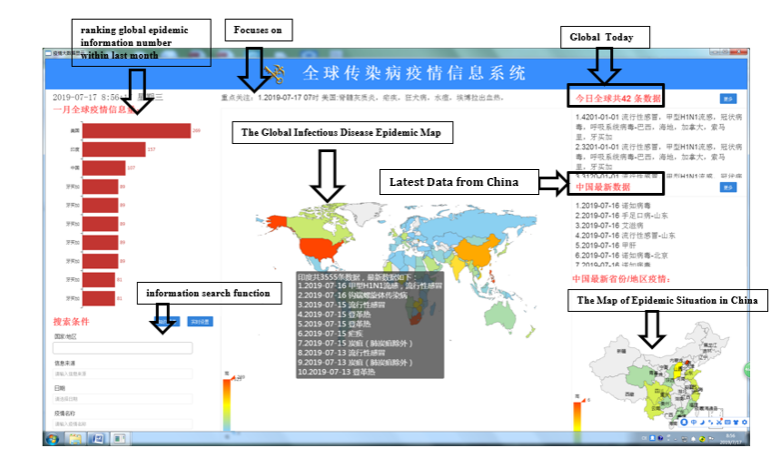
The Global Infectious Diseases Epidemic Information Monitoring System map.

The map has two submaps: (1) a global infectious disease epidemic map (GDM), and (2) a map of the epidemic situation in China (ESC) (Figure 2). The GDM presents the epidemic information for each country using different colors and can update the epidemic information for different countries in 3 seconds. The second map, the ESC, shows the epidemic information available for different regions of China, differentiated by color. When the mouse hovers over a region on the map, a list of the captured epidemic information from this region will be automatically displayed. By clicking on the list, users can obtain the relevant detailed information. For user interest, the map shows epidemic information by countries/regions, global today (list of global epidemic information available within the last 24 hours), latest data from China (list of domestic epidemic information available within the last month), ranking of the amount of global epidemic information available within the last month (ie, the amount of epidemic information available for each country, ranked from most to least), and an information search function.

The GIDEIMS map provides information and data services with simple interfaces and convenient operation. Travelers and travel health officers can simply identify the global human infectious disease epidemic situation at a glance.

#### Latest Outbreaks

In the latest outbreaks part of the system, the epidemic situation over the last 48 hours is shown, and information is updated hourly. Epidemic situations can be classified based on different data source websites and different kinds of diseases. At present, this mode is updated on an hourly basis. A large amount of data is intelligently analyzed using the background cloud server to automatically search and mine infectious disease epidemic information from official epidemic websites. The GIDEIMS can intelligently extract information including name of the epidemic disease, occurrence area, and epidemic description information from the searched webpages. Subsequently, the system automatically translates the data into Chinese and displays them to the users, facilitating the understanding of the epidemic information by users. The GIDEIMS also provides links to the original webpages and web snapshots to facilitate verification.

#### Epidemic Inquiry

The epidemic information collected by the GIDEIMS contains the resource titles, country/area, name of the infectious disease, reported date, crawling date, information sources, number of infected cases, number of deaths, and more. Users can utilize the general inquiry function to search the aforementioned epidemic information. The query results can be displayed in the format of lists or map reports. The outbreaks in different regions and countries can be viewed at a glance.

Users can access announcements from the health administrative department. These announcements contain the main introduction of disease, epidemic information, quarantine measures for travelers, advice for travelers heading to the area of the epidemic. Chinese travel health officers should follow the regulations of the administrative department—for example, a certain country with a severe COVID-19 epidemic situation has been regarded as a focus by the administrative department, and travelers from that country should receive COVID-19–related examinations when they enter China; hence, the prompt distribution of such announcements is very important for public health management.

The epidemic inquiry function contains an “important epidemic” section showing important infectious disease epidemic information, such as quarantine diseases that the Frontier Health and Quarantine Law (China) has regulated and other important epidemic diseases. Quarantine diseases include plague, cholera, and yellow fever, as stipulated by the state council of China [48]. In 2020, the essential epidemic diseases include plague, cholera, yellow fever, influenza, polio, Zika, dengue, chikungunya, malaria, measles, West Nile fever, and COVID-19. The list of diseases may be edited by the users in the basic setup section.

Based on the requirements set by the users, the number of infected cases and deaths, as well as epidemic information for areas of interest to the users, can also be accessed.

#### Other Functions

Other functions of the GIDEIMS are epidemic information entry, general analysis, basic setup, and further functions.

In the epidemic information entry function, travel health staff can manually enter epidemic information. The general analysis function reports epidemic information in a user-defined manner. Basic setup includes user management, important epidemic management, and important area management. For user management, the administrator may add, edit, or delete users. Also, criteria such as “important epidemic” or “important area” could be adjusted based on the user’s requirements.

The GIDEIMS is continuously upgraded because of many factors, such as users’ needs, source websites changes, or the global epidemic situation of a certain disease. Users will be able to send epidemic information to administrative departments and be linked to defined social media.

### Practical Case

In this section, we illustrate the GIDEIMS as a practical case to assess the system’s functionalities. The GIDEIMS map is seen by users via an independent path (ie, a virtual private network), while the other functions of the GIDEIMS are shown as websites.

Prior to using the GIDEIMS map, users install the specific application program provided by the developer in order to view the map (Figure 2). On July 16, 2019, the GIDEIMS collected 46 pieces of data compared with 10 pieces of data provided by the Global Incident Map and 6 pieces of data provided by Outbreak News Today (see Table 3). The data included 27 types of epidemic information from 23 countries/areas (10 websites). Two pieces of data were collected by both the Global Incident Map and Outbreak News Today (numbers 8 and 10 in Table 3).

**Table 3:** Epidemic information collected on July 16, 2019.

Data item number	Country/area	Title	Disease	Resource
1	DRC^a^	DRC: >2,500 cumulative cases of Ebola; 1,668 deaths [Chinese]^b^ [49]	Ebola	Baidu News
2	DRC	Fighting between DRC and Ebola has triggered the first confirmed case in Goma [Chinese]^b^ [50]	Ebola	Baidu News
3	DRC	DRC: Ebola virus disease [update] [Chinese Traditional]^b^ [51]	Ebola	Travel Health Service, Department of Health (Hong Kong, China)
4	DRC	Measles outbreak in the DRC [Chinese]^b^ [52]	Measles	Baidu News
5	DRC	WHO^c^ will take up Ebola emergency declaration question for a fourth time [53]	Ebola	CIDRAP^d^
6	Myanmar	Swine flu death toll rises to 54 in Myanmar [54]	Influenza	Global Incident Map
7	Pakistan	54 more dengue cases surface [55]	Dengue	Global Incident Map
8	Singapore	Singapore reports 666 dengue cases last week [56,57]	Dengue	Global Incident Map; Outbreak News Today
9	Singapore	Dengue cases: 75 cases notified 16 Jul 2019 at 3 pm [58]	Dengue	National Environment Agency, Singapore
10	Philippines	Dengue in Zamboanga up 285 percent this year [59]	Dengue	Global Incident Map; Outbreak News Today
11	Cameroon	Cholera kills five in Far North region [60]	Cholera	Global Incident Map
12	India	H1N1 flu on the rise, 309 cases in Mumbai [61]	Influenza	Global Incident Map
13	India	Leptospirosis—India (03): (Maharashtra) [62]	Leptospirosis	ProMED-mail
14	India	Japanese encephalitis & other—India (17): (AS) [63]	Japanese encephalitis	ProMED-mail
15	India	Nipah-affected student to be discharged on Tuesday [64]	Nipah virus	Global Incident Map
16	India	8-year-old dies due to suspected dengue fever [65]	Dengue	Global Incident Map
17	Bangladesh	Nearly 2,800 cases in first 16 days of July [66]	Dengue	Global Incident Map
18	Canada	Canada: 1st human rabies case reported since 2012 [67]	Rabies	Outbreak News Today
19	Canada	Canada: Syphilis outbreak in Alberta [68]	Syphilis	Outbreak News Today
20	United States	Cryptosporidium in the US with Joseph Eisenberg, PhD [69]	Cryptosporidiosis	Outbreak News Today
21	United States	Anthrax in Texas update: Eight premises in three counties [70]	Anthrax	Outbreak News Today
22	United States	Florida reports 1,900 hepatitis A cases so far, Pasco County has seen the most [71]	Hepatitis A	Outbreak News Today
23	United States	Angiostrongylus cantonensis—US (04): (HI) [72]	Angiostrongyliasis	ProMED-mail
24	United States	Infant botulism—US (02): (TX) more cases [73]	Botulism	ProMED-mail
25	United States	Undiagnosed respiratory illness—US (02): (VA) fatal, retirement community [74]	Undiagnosed	ProMED-mail
26	United States	El Paso reports 3rd measles case [75]	Measles	Outbreak News Today
27	United States	Rise in Candida auris cases; New AMR plan in Wales [76]	Candida auris	CIDRAP^d^
28	Malaysia	Malaria—Malaysia: Pahang, resurgence [77]	Malaria	ProMED-mail
29	Saudi Arabia	MERS-CoV (57): Saudi Arabia (NJ,RI) WHO [78]	MERS^e^	ProMED-mail
30	Saudi Arabia	WHO notes clusters in recent MERS cases, unveils environmental sampling guide [79]	MERS	CIDRAP
31	France	Salmonellosis—France: cured ham, alert, recall [80]	Salmonellosis	ProMED-mail
32	Syria	Brucellosis—Syria: (Quneitra) increasing incidence [Arabic]^b^ [81]	Brucellosis	ProMED-mail
33	Kenya	Anthrax—Kenya (12): (KU) human, cattle [82]	Anthrax	ProMED-mail
34	Taiwan	One newly diagnosed case of local dengue fever in Tainan [Chinese Traditional]^b^ [83]	Dengue	Taiwan CDC^f^
35	Taiwan	A new case of enterovirus complicated with severe illness [Chinese Traditional]^b^ [84]	Hand, foot and mouth disease	Taiwan CDC
36	Brazil, Cambodia, Pakistan, etc	Dengue fever or Chikungunya fever are prevalent in many countries in the world [Chinese Traditional]^b^ [85]	Dengue, chikungunya	Taiwan CDC
37	Europe	Rising European measles vaccination [86]	Measles	CIDRAP
38	Thailand	The disease control department recently revealed that the situation of dengue fever is slowing down [Thai]^b^ [87]	Dengue fever	The Department of Disease Control of Thailand
39	Nigeria	An update of Lassa fever outbreak in Nigeria for Week 27 [88]	Lassa fever	Nigeria CDC
40	Nigeria	An update of Cholera outbreak in Nigeria for Week 27 [89]	Cholera	Nigeria CDC
41	Namibia	H1N1 claims life in Windhoek [90]	Influenza	Global Incident Map
42	China	The reported incidence of hepatitis A and B in Gansu Province was significantly reduced [Chinese]^b^ [91]	Hepatitis A and B	Baidu News
43	China	Foodborne illness—China: Beijing, tap water, norovirus suspected [92]	Norovirus	ProMED-mail
44	China	Suspected Norovirus Infection in Residents of Wanke Qingqing Home District, Chaoyang, Beijing [Chinese]^b^ [93]	Norovirus	Baidu News
45	China	Shandong: In June, fewer people got the flu than those infected with hand, foot, and mouth disease [Chinese]^b^ [94]	Hand, foot, and mouth disease	Baidu News
46	Global	The latest report issued by UNAIDS: the mixed situation of countries [Chinese]^b^ [95]	HIV	Baidu News

^a^DRC: Democratic Republic of the Congo.

^b^Translated by Baidu Translate.

^c^WHO: World Health Organization.

^d^CIDRAP: Center for Infectious Disease Research and Policy.

^e^MERS: Middle East Respiratory Syndrome.

^f^CDC: Centres for Disease Control and Prevention.

**Figure 3:**
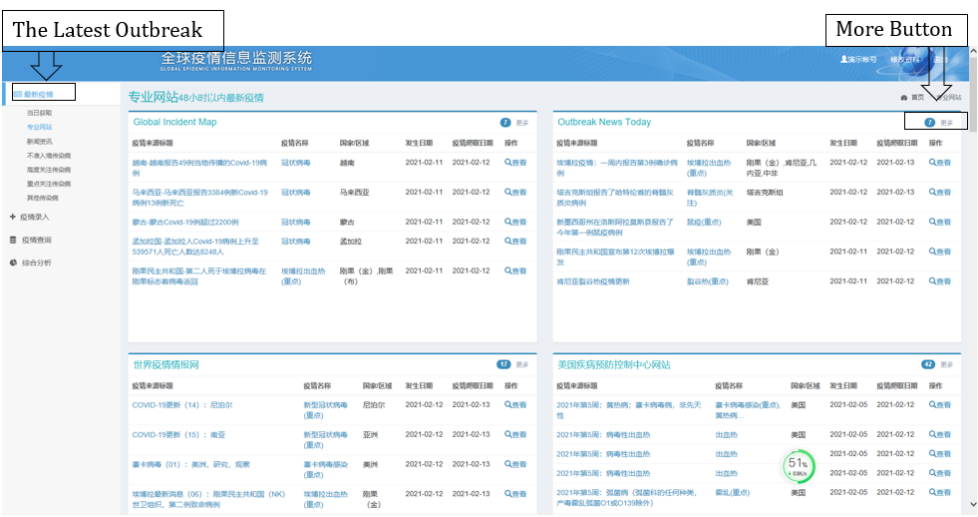
First page of the Global Infectious Diseases Epidemic Information Monitoring System, showing the latest outbreak.

**Figure 4:**
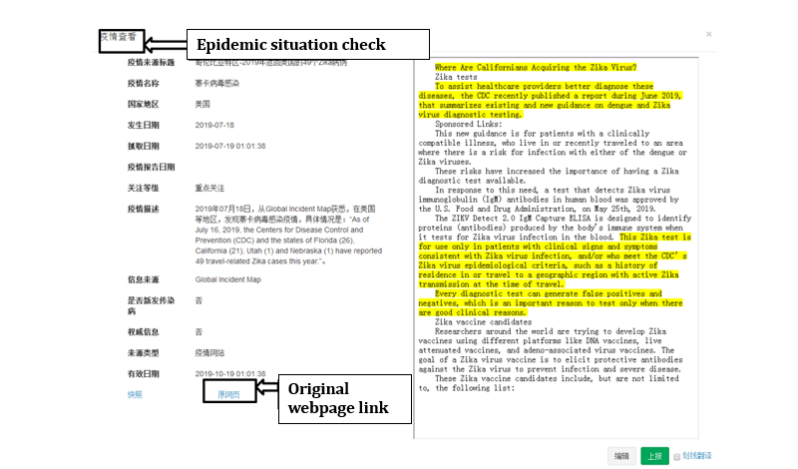
Detailed information provided by the Global Infectious Diseases Epidemic Information Monitoring System.

For the other functions of the GIDEIMS, first, the user must access the system using a defined website address [96]. After entering the username and password, the user sees the latest outbreak page (Figure 3), which shows the last five pieces of epidemic information news from each website. By clicking the “more” button, the user gains access to further epidemic information. The “check” button displays the detailed information (Figure 4). Users can click the “original webpage link” to verify the information.

**Figure 5:**
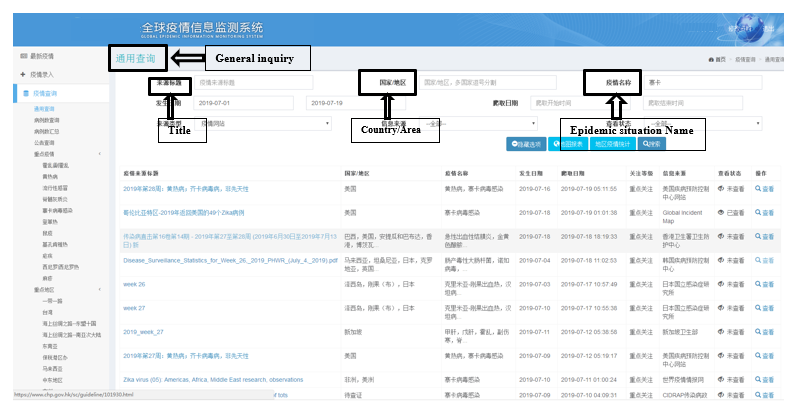
Sample inquiry about the Zika epidemic situation using the general inquiry function of the Global Infectious Diseases Epidemic Information Monitoring System.

Users wishing to search, for example, for recent Zika epidemic information would click “Epidemic inquiry,” followed by “General inquiry,” enter the epidemic disease name “Zika (in Chinese)” in “Epidemic situation name,” and limit the occurrence date to July 2019. The results are shown in Figure 5.

Shenzhen travel health officers use the epidemic information on a daily basis to guide their decision-making on infection disease epidemic management, such as identifying travelers with high fever and investigating their travel history. For travelers arriving from epidemic areas of certain infectious diseases, the officers will obtain a sample and test for the infectious diseases of interest if the traveler consents.

In contrast, without the GIDEIMS, staff could spend approximately 4 hours (excluding translation time) identifying the relevant epidemic information. With the use of the GIDEIMS, staff only need to access the collected data and verify them using the links and snapshots provided by the system when necessary. Working time is shortened to less than 1 hour. The information collected by the GIDEIMS is more accessible and comprehensive than that collected through the manual method.

## Discussion

Using the epidemic information provided by the GIDEIMS, health officers can quickly focus on travelers from certain countries and/or regions and discover infected cases as early as possible. By using the information from the GIDEIMS, staff at the Health Quarantine Center Laboratory of the Shenzhen International Travel Health Care Center (Shenzhen Customs District Port Outpatient Clinics) detected the first imported Zika cases in China [97], and thousands of suspected cases of certain infectious diseases have also been detected annually [98].Thus, while the detection work is strenuous, the GIDEIMS provides a helpful tool to effectively and sustainably identify suspected infected travelers into the Port of Shenzhen. The main advantage of the GIDEIMS is that it can automatically collect epidemic information from defined websites and translate it into the Chinese language. Although occasionally the system may duplicate information and require a manual check, most of the users of the GIDEIMS—such as the travel health officers working at Customs, travelers, researchers, and others working in the infectious disease control and prevention sector—reveal that the GIDEIMS saves time and is less labor-intensive. The first version of the GIDEIMS was developed in 2016 [99]. It is constantly upgraded to fulfill the requirements of users and adjusted according to actual situations. We constructed a visual display platform for the global infectious diseases epidemic information. The GIDEIMS is a user-friendly tool to support both travelers and travel health officers in travel health management. Meanwhile, big data obtained from the GIDEIMS may be used for infectious disease surveillance management and control.

In regard to COVID-19, information systems have largely been built to address almost every aspect of health management, including infection situation data management, remote health care system management, and syndromic surveillance [100-102]. In comparison, the GIDEIMS provides more comprehensive disease information than other COVID-19 epidemic information systems. Compared with the geographic information system for global monitoring of COVID-19 established by Johns Hopkins University [103], whose initial data were collected from the World Health Organization (WHO), US Centers for Disease Control and Prevention (CDC), China CDC, European Centre for Disease Prevention and Control (ECDC), National Health Commission of China (NHC), and DXY (a Chinese health-focused social website), the GIDEIMS could provide a full picture of the worldwide situation of the COVID-19 epidemic.

GIDEIMS breaks the barriers of language, region, time difference, and more so that it can provide enormous amounts of real-time infectious disease-related information. The system is not targeted on the individual traveler, but it provides travelers with useful information on the epidemic situation of infectious diseases.

At present, the GIDEIMS is a nonprofit application information system, where the maintenance and upgrade operations are performed by the developers free of charge. Due to the limitations of funds and human resources, the system is available in Chinese only. It is mainly provided to relevant infectious disease prevention and control departments and personnel for a free trial.

## Abbreviations

CDC: Centers for Disease Control and Prevention
ECDC: European Centre for Disease Prevention and Control
ESC: epidemic situation in China
GDM: global infectious disease epidemic map
GIDEIMS: Global Infectious Diseases Epidemic Information Monitoring System
MERS: Middle East Respiratory Syndrome
NHC: National Health Commission of China
WHO: World Health Organization
